# Disappearance of Symptoms of Spinal Atrophy Following Removal of the Ovaries and Fallopion Tubes

**Published:** 1885-01

**Authors:** 


### Disappearance of Symptoms of Spinal Atrophy Following Removal of the Ovaries and Fallopian Tubes.

Dr. Mundé reports the removal of the ovaries and fallopian tubes of a lady, 32 years old, suffering from symptoms of spinal atrophy for seven years. She had been bedridden, unable to walk, during this period. A vaginal examination showed retroversion of the uterus and a prolapsed, tender ovary. All efforts to relieve this condition failed. Oöphorectomy was suggested, but abandoned because of the small chances of relief promised thereby.

No relief coming to her by other treatment, she requested that the operation be done. After due deliberation and consultation, it was agreed upon to give the patient this small chance of relief. Four days after the operation, she moved the toes of the left foot, the first time in seven years. In one week she bent the left knee. In fifteen to seventeen days she began to learn again to walk. She did not drag the left foot, as formerly, in attempts to walk, but placed it directly forward. Two months after operation, she was able to walk unassisted the full length of a double room.—*New York Medical Journal.*

